# Supplementary material for: Patterns of Intron Gain and Loss in Fungi
Source: PLoS Biol. 2004 Nov 30;2(12):e422. doi: 10.1371/journal.pbio.0020422 (PMC532390; doi:10.1371/journal.pbio.0020422)
Supplement: Table S1 — Also available at http://genes.mit.edu/NielsenEtAl/. (4.3 MB ZIP). [file pbio.0020422.st001.zip › NielsenEtAl/html/496.html]

AN3740.1.NCU03605.1.MG08206.1.FG10131.1


```
 CLUSTAL W (1.82) Multiple Sequence Alignments - Introns Inserted


Sequence 1: NCU03605.1	359 aa
Sequence 2: MG08206.1	346 aa
Sequence 3: FG10131.1	346 aa
Sequence 4: AN3740.1	360 aa
Alignment Length: 378 aa
Number Identitical Residues: 115 aa
Alignment Score (without introns) 7166


MG08206.1 	MSST---DFPIVDSHIHLYPEAELETFKWYTPDNPLGKRHSLAEYRAATGSPSNLKGFVF
NCU03605.1	MASSPAVSVPIIDTHVHLYPQSEVESHQWYAEDNPLAKQHSIQEYQTATGAPGNLQGYVA
FG10131.1 	MSDD-QLILPIIDSHIHLYPASEVDSLSWYTPENPLAGQHSLEEYREATSSAPSLLGFVF
AN3740.1  	------MAFPIVDSHIHLFPESHLPTLAWYTPDNPLASQHSVDEYRSAVKSSTSLRGFIF
          	        .**:*:*:**:* :.: :  **: :***. :**: **: *. :. .* *:: 

MG08206.1 	LETDRLND------EGRDWKLPLMEVEWLARIVDGKPRDGEGHTPEDAPLCLGIVPWAPM
NCU03605.1	IEVDRKND------NAKDWTYPLQEIAWLRRIVTGQPKPGEGHTADDGRDCLGIVPWAPV
FG10131.1 	VETDRKNDVEAGAADGSGWEGPLAEVSWLKRIALGQPREGEGHTAEDAKLCLGIVPWAPV
AN3740.1  	LETDRLSSVEESETGKHGWTHALDEVSLLARIASGTPLPGEGHNAEDRDLCLGIVPWAPV
          	:*.** .. . . :   .*  .* *:  * **. * *  ****..:*   *********:

MG08206.1 	NLGAAKVEEYLEQAEKVAGP-ATWAKVKGFRYLLQDKPAASGLTEEFVESMRLLGKKGFI
NCU03605.1	VLGSAELEKYLAAAEKEAGP-ETWAKVKGFRYLLQDKPNGTALSDDFIEGLKLLGRKKFV
FG10131.1 	VSGPEVLERYLDRVKEEAG--DAWPKIKGFRYLLQDKPHGTMLDDKFIEGVKLLGRRGFT
AN3740.1  	SGGPDALEKYMALVKERAGSEEVWRKIRGVRYLVQDKPAGVMLQPAFIEGLKWLGRKGLT
          	  *.  :*.*:  .:: **.. .* *::*.***:**** .  *   *:*.:: **:: : 

MG08206.1 	FDAGVDQHRRGRQQLDELVSMIDQAHDGIANEDDKVKFIIN1HFCKPNLEIIN------P
NCU03605.1	FDLGVDQHKRGRIQLEEAVEMIDRAHEGVD-EEDKVVFILN1HLCKPDLTIVNV-----Q
FG10131.1 	FDVGVDQHRRGKKQLEEVVAFVERVHDGVP-EEEKVTLVLN1HLCKPDFSVINV-----T
AN3740.1  	FDLGVDARQGGIWQLEEAVEMMRRVYEGVE-EQKKVTIVIN1HLCKPNLRLQPTSEDSIT
          	** *** :: *  **:* * :: :.::*:  *:.** :::* *:***:: :   :..:  

MG08206.1 	ADPSFIAWRAAMFTLGKCDRVYMKLSGMFSEMPESLR--QRSMEEIFEAVQPWLAVVLAA
NCU03605.1	TDPSYIAWRTAMFTLSKCDRTYMKLSGCFSEMPEFLR--ARPAEEILSAILPWLAVVIAA
FG10131.1 	SDPGFHAWRTAVYALSKDSHVYMKLSGGFSEMPDSLR--KQDPSHIFEATLGWLGITLAT
AN3740.1  	THPDFTQWKTQVTAMAQYPATYMKLSGAFSELPPLSPGFESDIESVIDKLQPWTDAVFDA
          	:.*.:  *:: : ::.:   .****** ***:*    .     . ::.    *   .: :

MG08206.1 	FGPSRLMFASDWPVCTVGVDEEDAGGAWKKWKAVIDRLCRLASLSEEDQKMIWSGTAVKA
NCU03605.1	FGPERIMFGSDWPVCTLGLGDE----AWKKWRRVVEMTCHMAGLGPEAQTRLWAETAREA
FG10131.1 	FGPSRIMFGSDWPVCTAGTDNA-----WPRWRSIVERMCWMATLGDEERAMIFGGTAKKA
AN3740.1  	FGPERIMFGSDWPVCNIGGGGN--LVSWSRWNRVVTSVLERRGLDEKHSKGIWGQVAVKA
          	***.*:**.******. * .      :* :*. ::        *. :    ::. .* :*

MG08206.1 	YGIDI--------------
NCU03605.1	YKLDSRPVYKVVDLTRSDE
FG10131.1 	YNL----------------
AN3740.1  	YGVTLRDTRL---------
          	* :
```
